# Changes in cortical auditory evoked potentials in response to auditory training in elderly hearing aid users: A pilot study

**DOI:** 10.1371/journal.pgph.0000356

**Published:** 2022-05-03

**Authors:** Yara Bagali Alcântara, Willians Walace Fante Toledo, Karoline Ribeiro de Lima, Aline Tenório Lins Carnaúba, Eduardo Federighi Baisi Chagas, Ana Claudia Figueiredo Frizzo

**Affiliations:** 1 Speech Language Pathology Department, Faculty of Philosophy and Sciences, São Paulo State University—UNESP, Marília, São Paulo, Brazil; 2 Centro Universitário Cesmac, Maceió, Brazil; 3 Associated Graduate Program in Speech Language Pathology for the Federal University of Paraíba (UFPB), João Pessoa, Brazil; 4 Federal University of Rio Grande do Norte (UFRN), Natal, Brazil; 5 UNCISAL, Maceió, Brazil; 6 Postgraduate Program in Structural and Functional Interactions in Rehabilitation, University of Marília (UNIMAR), Marília, São Paulo, Brazil; 7 Postgraduate Program, Faculty of Medicine of Marília (FAMEMA), Marília, São Paulo, Brazil; Egas Moniz Cooperativa de Ensino Superior CRL, PORTUGAL

## Abstract

**Objective:**

To compare the cortical auditory evoked potential responses pre-and post-Auditory Musical Training associated with hearing aid adaptation in elderly people with presbycusis.

**Design:**

This is a pilot, prospective, randomized, single-blind study.

**Study sample:**

Eight presbiacusis elderly people between 65 and 80 years, new hearing aid users, divided into two groups participated in the study: Hearing Aid Group: use of hearing aid; and Auditory Training Group: use of hearing aid in addition to musical auditory training for 16 sessions. All participants were submitted to cortical auditory evoked potential tests with verbal stimulation in two different moments: Initial assessment, carried out before hearing aid adaptation and auditory training, and after three months, final assessment at the end of the auditory training sessions. All participants were adapted bilaterally with digital mini hearing aids.

**Results:**

There was a decrease in the P3a latency component for the Auditory Training Group when initial and final assessment were compared.

**Conclusion:**

There was a change in the cortical auditory evoked potential in elderly people with presbycusis in response to the Musical Auditory Training associated with the use of hearing aids in elderly people with presbycusis.

## Introduction

Hearing loss associated with aging—presbycusis—affects not only the peripheral auditory system; but changes in the anatomy and physiology of the central auditory system of the elderly can also occur, in addition to the changes caused by the aging process itself [1].

Even in mild hearing loss, a fundamental impact on the organization and functioning of the auditory cortex follows, as there is the recruitment of other sensory systems, such as the somatosensory system in an attempt to regulate the deficient neural processing of auditory information [2].

The impacts resulting from hearing deprivation in the elderly can be reduced with the appropriate intervention for hearing loss [2, 3].

Hearing rehabilitation may include hearing aid adaptation and auditory training, which is essential for the development of central auditory skills, such as auditory closure, auditory figure-ground, temporal ordering (duration pattern and frequency pattern), binaural separation and binaural integration [4].

The objective of auditory training, according to Bamford [5] is to develop or rehabilitate auditory skills, necessary for understanding sounds. Auditory training does not improve peripheral auditory sensitivity, that is, there is no change in tonal thresholds. However, this intervention proposal through auditory stimulation is directly related to brain reorganization through patients’ Central Auditory Nervous System (CANS) plasticity, improving attention and perception of more complex sounds, such as speech, allowing the individual to experience different sounds more clearly [6, 7].

Chermak and Musiek [4] state that in the elderly population the neural substrate is also subject to changes after auditory training, despite the plasticity potential reduction of the nervous system at this stage of life. For this reason, training strategies provide more compensation than recovery.

Musical Auditory Training (MAT) is an auditory training program developed to meet the needs of elderly hearing aid users, focused mainly on the auditory skills of temporal processing—resolution and temporal ordering—and selective attention. After the MAT application in elderly hearing aid users, an improvement in temporal auditory skills was found, proven by behavioral tests, evidencing the benefit of MAT in these skills [8–10]. However, this investigation found no studies that used electrophysiological measures to assess the effects of this auditory training program on the elderly’s auditory function.

The mechanism that enables this change in the auditory behavior, resulting from a neural reorganization, is neural plasticity [11]. The rehabilitation success through auditory training depends on whether this stimulation is effective at inducing cortical reorganization [12].

The electrophysiological measure is one of the means of investigating the functions of the central auditory pathways and has been an important instrument in monitoring the therapeutic evolution of the elderly in the auditory rehabilitation process [13, 14]. Cortical Auditory Evoked Potential (CAEP) is one of these measures and allows the assessment of cortical activities, more specifically the discrimination, integration and attention skills of the brain, involving the function of the thalamocortical and corticocortical auditory pathways, primary auditory cortex and associative cortical areas [15].

Decreased latency of auditory evoked potentials is understood as a neurophysiological correlate of neuronal plasticity. This can be observed even before a behavioral manifestation, as it depends on the integration between conscious perception and more central cognitive processes [16–19]. Other studies have demonstrated that neural plasticity is fundamental for the benefits obtained with auditory training, as it stimulates the neural structures related to the performance of the trained auditory skills, bringing direct benefits to the individuals [17, 20].

Considering the complexity of the factors that may be associated with hearing difficulties in the elderly, aging has been the subject of several studies in the area. Understanding the central auditory mechanisms involved in the auditory adaptation and rehabilitation process in this population is a challenge. Thus, this study seeks to investigate the therapeutic evolution at a central level in the elderly through Electrophysiology, more specifically the CAEP.

The hypothesis is that auditory rehabilitation through Musical Auditory Training (MAT) associated with the use of hearing aids is capable of generating changes in the auditory function in elderly people with presbycusis. If the hypothesis is confirmed, there may be a change in the clinical routine, favoring the indication of controlled auditory training programs, which added to the benefits obtained from the use of hearing aids can contribute to the full restoration of the hearing function of elderly people with difficulty in speech recognition in noise.

## Objective

To compare the cortical auditory evoked potential responses pre- and post-Auditory Musical Training associated with hearing aid adaptation in elderly people with presbycusis.

## Method

The present study was a pilot, prospective, randomized, single-blind study. It was carried out after approval by the Research Ethics Committee of the Faculty of Philosophy and Sciences–UNESP campus of Marília, under number 02848918.2.0000.5406, in accordance with Resolution 466/2012. To participate in the sample the patients signed a form of free and informed consent after being given information about the research.

### Subject selection and sample characterization

The sample selection of the study evaluated 22 individuals; 12 individuals were excluded, according to the exclusion criteria and two dropped out during the research.

Eight individuals participated in this study, divided into 2 groups (G) by random allocation:

Hearing Aid Group (HAG): composed of four individuals who had been recently adapted with hearing aids.

Auditory Training Group (ATG): composed of four individuals recently adapted with hearing aids and who participated in an auditory training program.

The inclusion criteria for participant selection were: age equal to or over 60 years; diagnosis of bilateral, symmetrical sensorineural hearing loss, with tonal audibility threshold between 30 and 70dB HL at frequencies of 3, 4, 6, and 8 kHz and ≤ 25 dB HL at frequencies of 0.25, 0.50, 1 and 2 kHz; percentage index of speech recognition equal to or over 60%, bilaterally; complaints of difficulty understanding speech in noise; hearing aid adaption referred by an otolaryngologist.

The exclusion criteria to rule out individuals were: presence of middle ear abnormality detected by tympanometry, when the tracings were not classified as type A, to ensure that sound transmission occurred without deficit in the peripheral auditory system; positive screening for cognitive impairment detected by the instrument Montreal Cognitive Assessment—Basic (MoCA-B), developed from the Montreal Cognitive Assessment (MoCA) original version, developed by Ziad Nasreddine and adapted to the Brazilian population by Daniel Apolinario [21] with the objective of distinguishing between normal cognitive aging and mild cognitive impairment; absence of wave V click-stimuli evoked during the Brainstem Auditory Evoked Potential (BAEP) examination for neurodiagnosis to ensure the integrity of the stimulus conduction to the central auditory nervous system and the absence of retrocochlear hearing loss; had used hearing aids before the study; and had undergone musical training.

The individuals who participated in the study were patients from a Specialized Rehabilitation Center, thus, the hearing rehabilitation mentioned in this study follows the procedures described in the Hearing Rehabilitation Instruction of the program “*S**aúde sem Limite”* by the National Health System from Brazil, which includes hearing aid adaptation and hearing rehabilitation [22].

The participants were new hearing aid users and were adapted bilaterally, with mini hearing aids, from the same manufacturer and with the same digital signal processing, with the same prescriptive rule NAL-NL2 and open adaptation technology, adjusted according to their individual audiograms. The gain of the hearing aids was verified through insertion gain, ensuring they were over 90% on target for all participants. The adaptation was completed in up to one month following the protocol mentioned above.

Some variables were considered to assess group homogeneity. They were age, sex, score obtained on the MoCA instrument, school level, time of hearing deprivation and average hours of using hearing aids daily. The average hours of hearing aid use was consulted in the data logging of manufacturer’s software. School level and duration of hearing deprivation were self-reported by the participants and were considered because the school level interferes with cognitive functions and hearing deprivation can cause functional consequences for the individual’s hearing, such as reduced speech recognition in noise and in cognitive skills functions that contribute to speech comprehension, such as working memory and speed in processing acoustic information [23].

### Electrophysiological assessment

The CAEP was performed using the two-channel equipment Biologic’s Evoked Potential System (EP) (Seattle, WA/USA), and free-field system FF-70 with adapter compatible with evoked potential system was calibrated according to the ANSI S3.43 1992 / ISO 7566 and ANSI S3.6 1996/ISO R389 parameters.

The potential was recorded using five copper electrodes, positioned in: Fpz the ground electrode, Fz and Cz, which were the active electrodes, in reference to electrodes A1 and A2. Each electrode impedance did not exceed 5 k ohms and did not exceed 2k ohms between them. In order to obtain wave reproducibility in a simultaneous recording of Cz and Fz, ipsilateral assembly was performed using the jumper at A2 and then at A1.

The Table 1 below shows the parameters used for the CAEP capture.

**Table 1:** Summary of parameters used for the CAEP capture.

	Parameter	
Stimulus	Mode	Bilateral—sound field (speaker positioned at one meter and 0° azimuth—centralized)
	Rate	0.9 stimuli per second
	Type	Speech: /ba/ frequent; /da/ rare
	Intensity	20 to 25 dB SL, minimum 80 dB, not over 85 dB
	Polarity	Alternated
Acquisition	Channel	2 channel
	Electrode	Fz (+), A1 (-)
		Cz (+), A2 (-)
		Jumper connected to reference inputs (-)
	Filter	0,1–30 Hz
	Signal Amplification	50.000 times
	Sampling	200 stimuli* - 80% frequent and 20% rare
	Electrode Impedance	≤ 5 Kohms
	Patient condition	Alert ignoring stimulus

* The considered number of artifacts from the total stimulus was up to 10%.

The CAEP procedure was performed on the research patients in two moments: initial assessment and reassessment after three months of hearing aid adaptation. The individuals did not use hearing aids at the time of the test. During the interval between the two assessments, ATG underwent auditory training.

Latencies and amplitudes of the P1-N1-P2-N2-P3a wave complex were analyzed. As the test was performed passively, that is, participants were not requested to identify the rare stimulus, the P3a component was analyzed, which occurs automatically in response to major differences in stimuli, whether or not the individual is responding to the stimulus sequence [24, 25].

Considering the best wave morphology, the marking was performed on the infrequent Cz trace, and the P1-N1-P2-N2 components were marked and identified in the appearance of the first three waves, at the highest peak, in sequence, in the negative—positive—negative polarities, respectively, between 50 ms and 300 ms, and the P3a component was identified after the P1-N1-P2-N2 complex, between 220 ms and 400 ms [24].

The lead researcher was responsible for the auditory training application. Electrophysiological assessments were carried out by a second invited examiner, to ensure the groups were not identified and to eliminate possible detection bias. The wave marking was carried out by two expert researchers in the area.

### Auditory training

For the auditory training, the program *Treinamento Musical Auditivo*, developed by Dr. Katya Guglielmi Marcondes Freire was used, available on the website www.treinamentoauditivomusical.com.br [10]. The website also has an English version of the program. The program has eight exercises that contemplate figure-ground aspects of instrumental sounds, sound frequency and duration, directed listening, rhythm, auditory closure and memory. Each exercise has levels of difficulty to stimulate and challenge the auditory system. Each level of difficulty consists of ten exercises and the author’s recommendation is that the level of difficulty should increase when the patient obtains at least seven correct answers. Each exercise was performed in two sessions, according to the following presentation order: (1) figure-ground—instrumental sounds; (2) figure-ground—sequential sounds; (3) directed listening; (4) sound duration; (5) sound frequency; (6) rhythm, (7) auditory closure; (8) audio-visual memory. MAT was applied to ATG participants in 30-minute sessions, twice a week for eight weeks totaling 16 sessions. The 24-hour interval between sessions was respected, taking into account the importance of sleep in the consolidation of auditory perceptual learning [26]. Two portable speakers coupled to a computer were used in a sound-attenuated room, at one-meter distance between them and the patient, with one speaker positioned to the left and the other one to the right of the computer, at 45° Azimuth in relation to the patient. At the beginning of all sessions, the hearing aids were checked for functioning and the hearing aid setting was maintained during the training sessions, with an adaptive directional microphone and algorithms on, in order to maintain the listening conditions the participant would be routinely exposed.

### Statistical analysis

To analyze the main group and time effect, and the group and time interaction, a mixed ANOVA of repeated measures was performed based on the equality of covariance matrices through the Box’s Test. Post-Hoc comparisons within the group and within time were performed through the Holm-Sidak test. The adopted significance level was 5% (p-value ≤ 0.05), and the data were analyzed using the SPSS software (version 24.0). The values are described by the mean and standard deviation (SD).

## Results

Following, Table 2 shows the descriptive data for HAG and ATG participants to characterize the sample.

**Table 2:** Sample profile.

Participant	Group	Age	Sex	School level	MoCA Score (points)	Deprivation Time (years)	Mean of HA use (hours)
1	HAG	68	M	complete elementary school	27	5	12
2	HAG	72	F	complete elementary school	29	4	8
3	HAG	69	M	incomplete elementary school	26	5	9
4	HAG	65	M	complete high school	29	3	7
5	ATG	68	F	incomplete elementary school	26	3	7
6	ATG	70	F	incomplete elementary school	26	1	9
7	ATG	71	M	complete high school	28	2	8
8	ATG	80	F	incomplete elementary school	25	2	8

HAG = Hearing Aid Group; ATG = Auditory Training Group; M = male; F = female; MoCA = Montreal Cognitive Assessment; HA = Hearing Aid.

Based on these data, the statistical analysis was performed to verify group homogeneity, as shown in Table 3.

**Table 3:** Group comparative analysis.

Variable	Category	HAG	ATG			
		(n = 4)	(n = 4)			
		*F*	%	*F*	%	p-value
Age	60 to 70 years	3	75	2	50	0.999
	over 71 years	1	25	2	50	
Sex	Female	1	25	3	75	0.486
	Male	3	75	1	25	
School level	Complete elementary	3	75	1	25	0.486
	Incomplete elementary	1	25	3	75	
MoCA Score (points)	Between 24–27 points	2	50	3	75	0.999
	Over 28 points	2	50	1	25	
Deprivation Time (years)	Up to 3 years	1	25	4	100	0.143
	between 4–5 years	3	75	0	0	
Mean of HA use (hours)	Up to 8 hours daily	2	50	3	75	0.999
	Over 12 hours daily	2	50	1	25	

HAG = Hearing Aid Group; ATG = Auditory Training Group; MoCA = Montreal Cognitive Assessment; HA = Hearing Aid.

Note: * p <0.05 significant association by Fisher’s exact test.

Table 4 shows the comparison between the groups for the CAEP responses from the mean latencies of the P1-N1-P2-N2-P3a complex for the two moments of assessment, and the ANOVA results.

**Table 4:** Mean and standard deviation of the cortical auditory evoked potential latency values (ms) at the two assessed moments.

	Hearing Aid Group		Auditory Training Group (n = 4)		p-value		
	(n = 4)						
	Mean (ms)	SD	Mean (ms)	SD	Group	Time	Interaction
P1 Initial As	61.42	12.58	68.71	14.60	0.709	0.679	0.645
P1 Final As	61.73	24.70	62.98	16.71			
N1 Initial As	85.89	14.44	105.41	15.38	0.697	0.653	0.062
N1 Final As	104.88	18.16	92.91	16.98			
P2 Initial As	135.07	2.74	158.24	36.88	0.692	0.102	0.064
P2 Final As	137.67	17.45	125.18	11.82			
N2 Initial As	170.21	13.40	215.19	54.74	0.346	0.097	0.074
N2 Final As	172.81	11.05	160.58	12.36			
P3a Initial As	248.02	14.95	265.98	35.43	0.280	0.017†	0.848
P3a Final As	224.08	19.39	238.91*	12.68			

n = number of individuals; SD = standard deviation; As = assessment.

Note: Multivariate analyzes were based on the assumption of equality of covariance matrices through the Box’s test.

* Significant difference in relation to the initial assessment within the group through the Holm-Sidak Post-hoc test for p-value ≤ 0.05.

† main effect of time within the group through mixed ANOVA of repeated measures for p-value ≤ 0.05.

There was a statistically significant difference for the P3a component in the ATG when initial and final assessment were compared, indicating a time effect within the group, with a latency decrease in this component after hearing rehabilitation.

Table 5 shows the means of the amplitudes of the P1-N1-P2-N2-P3a complex for the two moments of assessment, and ANOVA results.

**Table 5:** Mean and standard deviation of the cortical auditory evoked potential amplitude values (μV) at the two assessed moments.

	Hearing Aid Group (n = 4)		Auditory Training Group (n = 4)		p-value		
	Mean (μV)	SD	Mean (μV)	SD	Group	Time	Interaction
P1 Initial As	1.05	1.13	2.06	1.16	0.087	0.122	0.126
P1 Final As	1.06	0.84	3.25	1.56			
N1 Initial As	-1.85	0.98	-3.06	2.58	0.090	0.401	0.938
N1 Final As	-0.72	0.76	-2.11	2.39			
P2 Initial As	0.79	0.86	3.60	2.59	0.143	0.397	0.058
P2 Final As	1.69	0.43	1.55	0.77			
N2 Initial As	-1.48	1.28	-3.12	2.74	0.151	0.453	0.814
N2 Final As	-1.93	1.96	-3.96	1.51			
P3a Initial As	2.49	1.25	3.15	1.44	0.291	0.295	0.354
P3a Final As	2.58	0.44	4.51	3.05			

n = number of individuals; SD = standard deviation; As = assessment.

Note: Multivariate analyzes were based on the assumption of equality of covariance matrices by the Box’s test.

There was no statistically significant difference in relation to the amplitudes of the P1-N1-P2-N2-P3a complex considering the analysis of the group and time effect, and the interaction between group and time.

**Fig 1:**
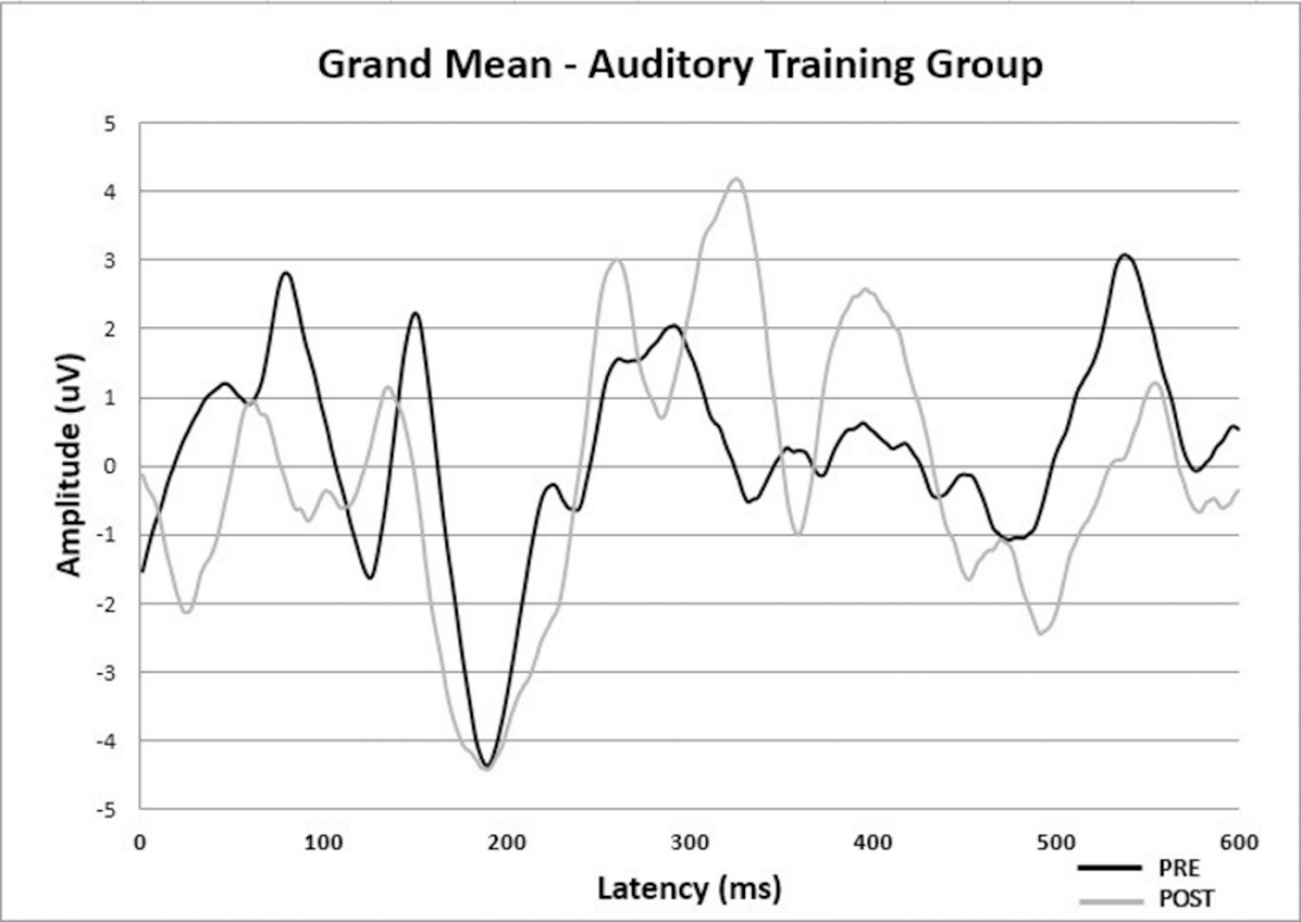
Cortical auditory evoked potential responses grand average at pre-and post-assessment of auditory training group.

Fig 1 shows the CAEP grand average of ATG pre-and post-Auditory Musical Training associated with hearing aid fitting to improve visibility of the results described in Tables 4 and 5. Predominantly for the statistically significant difference, it is possible to observe a decreased latency of what would be the P3a component in the post-assessment in relation to the pre-assessment, close to 300 ms in Fig 1.

## Discussion

Auditory rehabilitation process involves several factors that can interfere with the successful hearing aid adaptation and the benefits obtained with hearing training programs. Among them, the time of hearing deprivation, adherence to the use of hearing aids, among other factors that influence each case toward a different prognosis and require careful evaluation by the involved professionals [27].

Considering these factors, it is important to initially reckon the characteristics of the study sample. The groups were homogeneous in relation to age, education level, score obtained on the MoCA instrument, time of hearing deprivation and average hours of using hearing aids daily. There are no significant differences between them. In general, most of the elderly participants in the study were between 60 and 70 years old, had good cognitive level, time of hearing deprivation of up to 5 years, and systematic hearing aid use.

However, we must consider that the use of Fisher’s exact test in Table 3, a pilot study with a small sample, may suggest the probability that the HAG group has a longer period of auditory deprivation. Thus, this group could present more marked difficulties resulting from this longer period of hearing deprivation. Regarding the hours of hearing aid use, the probability of difference between the groups is lower. It is a limitation of Fisher’s exact test for small samples and should be considered in future studies.

Assuming that AEPs provides rich information for reasoning and understanding the neuroelectrophysiological processes that are part of auditory and linguistic information processing, this investigation chose to assess the CAEP. This measure investigates the detection, attention, discrimination and recognition of sound automatically, without the need for active patient participation, which minimizes the negative interference of factors related to age, such as memory and cognitive processes [28].

Some studies report Long Latency Auditory Evoked Potentials (LLAEP) increased latency in the elderly [29, 30] when compared to young people and adults. However, in the present study, latency values within the reference intervals were observed. Even though comparison with young patients was not performed, the elderly in both groups had good cognitive levels and time of hearing deprivation of up to five years. Another study showed that 2–5 year-time of hearing deprivation in individuals with mild or moderate sensorineural hearing loss did not influence the results of the LLAEP [31]. Another study assessed N1 and P2 latencies in elderly people with hearing loss from 4KHz frequency and complaints of speech comprehension, and observed no increased latencies for these components [32].

The comparison between the measures obtained in the initial and final assessments between the HAG and ATG identified a positive effect of auditory training for the elderly in the ATG, with a significant decrease in the latency of the P3a component after hearing rehabilitation compared to the values ​​of the initial assessment. For the other components of the wave complex, a decrease in latency was observed in the final assessment in ATG, but no statistically significant differences.

When latency decreases are observed after interventions, we can understand that the time of processing the acoustic information by CANS was reduced [33, 34]. Specifically for the P3a component, the reduction in latency shows improvement in attention, acoustic recognition and auditory-linguistic association (secondary auditory cortex and associative areas) after the musical auditory training sessions.

The P3a component reflects the automatic process of attention and perception of the distinctive acoustic characteristics of the rare stimulus during the CAEP examination. Studies show that P3a and P3b have different neural generators, and that P3a originates from frontal attention mechanisms [35]. A study carried out using the Low-Resolution Electromagnetic Tomography technique showed that P3a generators are located in the cingulate areas, frontal and parietal regions [36].

Studies involving auditory evoked potentials describe decreased latencies as a neurophysiological correlate of neural plasticity [16, 18]. Changes in P3 latency were observed in other studies involving elderly people who only used hearing aids [11, 37, 38], unlike our study, which did not observe significant changes for HAG.

Other studies assessing the effects of AT with electrophysiological measures showed an improvement in the neural representation of speech in noise after training [14, 39]. These studies did not associate the use of hearing aids with auditory training.

Some studies report P3 amplitude increase after auditory training [37, 40]. In this study, an increase in P3a amplitude was observed after auditory training, with no statistically significant difference.

The amplitude represents the magnitude of electrical activity involved in performing a task. When an increase in amplitude after interventions is observed, it is understood that there was activation of synaptic activity and an improvement in processing quality. And related to the P3a component, it reflects this activation for the process of redirecting attention to the automatic detection of the relevant acoustic stimulus [41].

The present study analyzed the CAEP measurements in sound field, in order to assess the neural responses to binaural hearing, considering its importance for the ability to recognize speech in noise in elderly people and your auditory and communicative decline related to age.

The sample size can be considered a limiting factor to this study, whose initial proposal was to have more participants in the groups. However, such results aroused the researchers’ interest in continuing the study to promote longitudinal assessments and to investigate long-term effects.

## Conclusion

There was a decrease in the latency of the P3a component of the Cortical Auditory Evoked Potential in the comparison pre- and post-Auditory Musical Training associated with hearing aid adaptation in elderly people with presbycusis.

## References

[1] Howarth A, Shone GR. Ageing and the auditory system. Postgrad Med J. 2006;82(965):166–171. doi: 10.1136/pgmj.2005.039388 16517797 PMC2563709

[2] Cardon G, Sharma A. Somatosensory Cross-Modal Reorganization in Adults With Age-Related, Early-Stage Hearing Loss. Front Hum Neurosci. 2018;12:172. Published 2018 May 3. doi: 10.3389/fnhum.2018.00172 29773983 PMC5943502

[3] Sharma A, Glick H, Campbell J, Torres J, Dorman M, Zeitler DM. Cortical Plasticity and Reorganization in Pediatric Single-sided Deafness Pre- and Postcochlear Implantation: A Case Study. Otol Neurotol. 2016 Feb;37(2):e26–34. doi: 10.1097/MAO.0000000000000904 26756152 PMC6530986

[4] Chermak GD, Musiek FE. Auditory training: Principles and approaches for remediating and managing auditory processing disorders. Seminars in Hearing. 2002 Nov;23(4):297–308. 10.1055/s-2002-35878 PMC4910543 27587909

[5] Bamford J. Auditory Training what is it, what is it supposed to do, and does it do it? British Journal of Audiology. 1981. 15:2, 75–78. doi: 10.3109/03005368109081418 7225653

[6] Musiek FE, Baran JA, Schochat E. Selected management approaches to central auditory processing disorders. Scand Audiol Suppl. 1999;51:63–76. . 10803915

[7] Tremblay KL, Billings CJ, Friesen LM, Souza PE. Neural representation of amplified speech sounds. Ear Hear. 2006 Apr;27(2):93–103. doi: 10.1097/01.aud.0000202288.21315.bd . 16518138

[8] Lessa AH, Hennig TR, Costa MJ, Rossi AG. Results of auditory rehabilitation in elderly users of hearing aids evaluated by a dichotic test. Codas. 2013; 25(2):169–75. doi: 10.1590/s2317-17822013000200013 . 24408247

[9] Hennig TR, Costa MJ, Rossi AG, de Moraes AB. Auditory rehabilitation effects on the temporal ordering ability in elderly hearing aids users. *J*. *Soc*. *Bras*. *Fonoaudiol*. 2012. 24, 26–33. doi: 10.1590/s2179-64912012000100006 22460369

[10] Freire KGM, Iorio MCM. Treinamento auditivo musical: uma proposta para idosos usuários de próteses auditivas. D.Sc. Thesis, Federal University of Sao Paulo. 2009. Available from: http://repositorio.unifesp.br/handle/11600/10143

[11] Musiek FE, Berge BE. A neuroscience view of auditory processing disorders. In: Central auditory processing disorders: mostly management. Boston: Allyn & Bacon; 1998 a. pp 15–32.

[12] Dias KZ, Gil D. Treino auditivo formal nos distúrbios de processamento auditivo. In: Tratado de Audiologia. Santos: Santos Editora; 2011. pp 829–844.

[13] Figueiredo SSR, Boechat EM. Privação e plasticidade sensorial auditiva em idosos: potenciais corticais e questionários de autoavaliação. Estud. Interdiscip. sobre o Envelhec. 2016. 21, 105–126.

[14] Anderson S, White-Schwoch T, Parbery-Clark A, Kraus N. Reversal of age-related neural timing delays with training. Proc Natl Acad Sci U S A. 2013 Mar 12;110(11):4357–62. doi: 10.1073/pnas.1213555110 Epub 2013 Feb 11. ; PMCID: PMC3600492. 23401541 PMC3600492

[15] Kraus N, Nicol T. Aggregate neural responses to speech sounds in the central auditory system. Speech Commun. 2003. 41, 35–47. doi: 10.1016/S0167-6393(02)00091-2

[16] Kraus N, McGee T. Potenciais Evocados Auditivos de Longa Latência. in Tratado de Audiologia Clínica. ed. Katz J. 1999. pp 403–414.

[17] Musiek FE, Berge BE. How electrophysiologic tests of central auditory processing influence management. In: BESS F. H. (Ed.) Children with hearing impairment: contemporary trends. Nashville: Vanderbilt Bill Wilkerson Center Press, 1998 b. pp 145–162.

[18] Russo NM, Nicol TG, Zecker SG, Hayes EA, Kraus N. Auditory training improves neural timing in the human brainstem. Behav Brain Res. 2005 Jan 6;156(1):95–103. doi: 10.1016/j.bbr.2004.05.012 . 15474654

[19] Tremblay K, Kraus N, McGee T. The time course of auditory perceptual learning: neurophysiological changes during speech-sound training. Neuroreport. 1998 Nov 16;9(16):3557–60. doi: 10.1097/00001756-199811160-00003 . 9858359

[20] Zalcman TE, Schochat E. A eficácia do treinamento auditivo formal em indivíduos com transtorno de processamento auditivo. Revista da Sociedade Brasileira de Fonoaudiologia, 2007. 12, n. 4, 310–314. doi: 10.1590/S1516-80342007000400010

[21] Apolinário D. Montreal Cognitive Assessment—Basic (MoCA-B) Instruções para Aplicação e Pontuação. 2015.

[22] Brasil. Ministério da Saúde. *Instrutivos de Reabilitação Auditiva, Física, Intelectual e Visual (CER e serviços habilitados em uma única modalidade)*. 2017. Available from: https://saude.rs.gov.br/upload/arquivos/carga20171010/13131007-portaria-793.pdf.

[23] Wong PC, Jin JX, Gunasekera GM, Abel R, Lee ER, Dhar S. Aging and cortical mechanisms of speech perception in noise. Neuropsychologia. 2009 Feb;47(3):693–703. doi: 10.1016/j.neuropsychologia.2008.11.032 Epub 2008 Dec 13. ; PMCID: PMC2649004. 19124032 PMC2649004

[24] Hall JW. New Handbook for Auditory Evoked Responses. Pearson Education; 2006.

[25] McPherson DL. Late potentials of the auditory system. Singular Publishing Group; 1996.

[26] Alain C, Zhu KD, He Y, Ross B. Sleep-dependent neuroplastic changes during auditory perceptual learning. Neurobiol Learn Mem. 2015 Feb;118:133–42. doi: 10.1016/j.nlm.2014.12.001 Epub 2014 Dec 6. . 25490057

[27] Tremblay KL. Central auditory plasticity. The Hearing Journal: 2003 Jan; 56(1). pp 10–15. doi: 10.1097/01.HJ.0000292995.53429.6a

[28] Tremblay KL, Piskosz M, Souza P. Effects of age and age-related hearing loss on the neural representation of speech cues. Clin Neurophysiol. 2003 Jul;114(7):1332–43. doi: 10.1016/s1388-2457(03)00114-7 . 12842732

[29] Gürkan S, Durankaya SM, Mutlu B, İşler Y, Uzun YÖ, Başokçu O, et al. Comparison of Cortical Auditory Evoked Potential Findings in Presbycusis with Low and High Word Recognition Score. J Am Acad Audiol. 2020 Jun;31(6):442–448. doi: 10.3766/jaaa.19063 Epub 2020 Aug 3. . 31914374

[30] Tremblay KL, Piskosz M, Souza P. Aging alters the neural representation of speech cues. Neuroreport. 2002 Oct 28;13(15):1865–70. doi: 10.1097/00001756-200210280-00007 . 12395081

[31] Bruckmann M, Didoné DD, Garcia MV. Privação sensorial auditiva e sua relação com os potenciais evocados auditivos de longa latência. Distúrbios da Comun. 2018. 30, 43.

[32] Cóser MJS, Cioquetta E, Pedroso FS, Cóser PL. Cortical Auditory Evoked Potentials in Elderly with Difficulty in Speech Understanding Complaint. Int. Arch. Otorhinolaryngol. 2007.

[33] Escera C, Alho K, Winkler I, Näätänen R. Neural mechanisms of involuntary attention to acoustic novelty and change. J Cogn Neurosci. 1998 Sep;10(5):590–604. doi: 10.1162/089892998562997 . 9802992

[34] Squires NK, Squires KC, Hillyard SA. Two varieties of long-latency positive waves evoked by unpredictable auditory stimuli in man. Electroencephalogr Clin Neurophysiol. 1975 Apr;38(4):387–401. doi: 10.1016/0013-4694(75)90263-1 . 46819

[35] Polich J. Updating P300: an integrative theory of P3a and P3b. Clin Neurophysiol. 2007 Oct;118(10):2128–48. doi: 10.1016/j.clinph.2007.04.019 Epub 2007 Jun 18. ; PMCID: PMC2715154. 17573239 PMC2715154

[36] Volpe U, Mucci A, Bucci P, Merlotti E, Galderisi S, Maj M. The cortical generators of P3a and P3b: a LORETA study. Brain Res Bull. 2007 Jul 12;73(4–6):220–30. doi: 10.1016/j.brainresbull.2007.03.003 Epub 2007 Apr 3. . 17562387

[37] Miranda EC. Estudo eletrofisiológico e comportamental da audição em idosos com alteração cognitiva antes e após a adaptação de próteses auditivas. D.Sc. Thesis, Federal University of Sao Paulo. 2012. Available from: http://repositorio.unifesp.br/handle/11600/21916.

[38] Rao A, Rishiq D, Yu L, Zhang Y, Abrams H. Neural Correlates of Selective Attention With Hearing Aid Use Followed by ReadMyQuips Auditory Training Program. Ear Hear. 2017 Jan/Feb;38(1):28–41. doi: 10.1097/AUD.0000000000000348 . 27556531

[39] Anderson S, White-Schwoch T, Choi HJ, Kraus N. Training changes processing of speech cues in older adults with hearing loss. Front Syst Neurosci. 2013 Nov 28;7:97. doi: 10.3389/fnsys.2013.00097 ; PMCID: PMC3842592. 24348347 PMC3842592

[40] Alonso R. Avaliação eletrofisiológica e comportamental do processamento auditivo (central) e treinamento auditivo em indivíduos idosos. D.Sc. Thesis, University of São Paulo. 2011. doi: 10.11606/T.5.2012.tde-16032012-092210

[41] Frizzo ACF, Advíncula KP. Potenciais Evocados Auditivos de Longa Latência: conceitos e aplicações clínicas. In Tratado de Eletrofisiologia para a Audiologia. BookToy; 2018. pp 139–150.

